# BPIFB4 and its longevity-associated haplotype protect from cardiac ischemia in humans and mice

**DOI:** 10.1038/s41419-023-06011-8

**Published:** 2023-08-15

**Authors:** Monica Cattaneo, Aneta Aleksova, Alberto Malovini, Elisa Avolio, Anita Thomas, Valeria Vincenza Alvino, Michael Kilcooley, Marie Pieronne-Deperrois, Antoine Ouvrard-Pascaud, Anna Maciag, Gaia Spinetti, Sophie Kussauer, Heiko Lemcke, Anna Skorska, Praveen Vasudevan, Stefania Castiglione, Angela Raucci, Robert David, Vincent Richard, Antonio Paolo Beltrami, Paolo Madeddu, Annibale Alessandro Puca

**Affiliations:** 1 Cardiovascular Department, IRCCS MultiMedica, Milan, Italy; 2 Cardiothoracovascular Department, Azienda Sanitaria Universitaria Giuliano Isontina, Trieste, Italy; 3 Laboratory of Informatics and Systems Engineering for Clinical Research, Istituti Clinici Scientifici Maugeri IRCCS, Pavia, Italy; 4 Translational Health Sciences, Bristol Medical School, University of Bristol, Bristol, UK; 5 Inserm U1096 EnVI, Rouen Normandy University, Rouen, France; 6 Department of Cardiac Surgery, Rostock University Medical Center, Rostock, Germany; 7 Faculty of Interdisciplinary Research, Department Life, Light & Matter, University Rostock, Rostock, Germany; 8 Experimental Cardio-oncology and Cardiovascular Aging Unit Centro Cardiologico Monzino, Milan, Italy; 9 Department of Medicine, University of Udine, Academic Hospital of Udine, ASUFC, Udine, Italy; 10 Department of Medicine, Surgery and Dentistry, University of Salerno, Salerno, Italy

**Keywords:** Myocardial infarction, Drug delivery

## Abstract

Long-living individuals (LLIs) escape age-related cardiovascular complications until the very last stage of life. Previous studies have shown that a Longevity-Associated Variant (LAV) of the BPI Fold Containing Family B Member 4 (*BPIFB4*) gene correlates with an extraordinarily prolonged life span. Moreover, delivery of the *LAV-BPIFB4* gene exerted therapeutic action in murine models of atherosclerosis, limb ischemia, diabetic cardiomyopathy, and aging. We hypothesize that downregulation of BPIFB4 expression marks the severity of coronary artery disease (CAD) in human subjects, and supplementation of the *LAV-BPIFB4* protects the heart from ischemia. In an elderly cohort with acute myocardial infarction (MI), patients with three-vessel CAD were characterized by lower levels of the natural logarithm (Ln) of peripheral blood BPIFB4 (*p* = 0.0077). The inverse association between Ln BPIFB4 and three-vessel CAD was confirmed by logistic regression adjusting for confounders (Odds Ratio = 0.81, *p* = 0.0054). Moreover, in infarcted mice, a single administration of *LAV-BPIFB4* rescued cardiac function and vascularization. In vitro studies showed that LAV-BPIFB4 protein supplementation exerted chronotropic and inotropic actions on induced pluripotent stem cell (iPSC)-derived cardiomyocytes. In addition, LAV-BPIFB4 inhibited the pro-fibrotic phenotype in human cardiac fibroblasts. These findings provide a strong rationale and proof of concept evidence for treating CAD with the longevity BPIFB4 gene/protein.

## Introduction

Coronary artery disease (CAD) and stroke remain the leading causes of morbidity and mortality in Western countries [1]. Three-vessel is the most severe and fatal form of CAD characterized by critical stenosis in the left anterior descending artery, the left circumflex artery, and the right coronary artery [2]. Patients with three-vessel CAD have a higher risk of death and major adverse cardiac events [3].

Unhealthy lifestyles and accrual of risk factors contribute to vascular dysfunction highlighted by cellular senescence and impaired synthesis and secretion of endothelium-derived vasoactive molecules [4–7]. Genetic factors also participate in determining the dichotomy between cardiovascular health and disease. Nonetheless, very few gene polymorphisms proved to capture the divergence of cardiovascular clocks seen in high-risk individuals (HRIs) and long-living individuals (LLIs). Among them, the longevity variant (*LAV*) of the BPI Fold Containing Family B Member 4 (*BPIFB4*) gene, showed a preponderant impact on the cardiovascular system and prolonged life span, passing the validation of three geographically unrelated cohorts. Carriers of the *LAV-BPIFB4* gene express high levels of the encoded protein in the blood, circulating mononuclear cells, and vascular cells [8–11]. Moreover, high levels of circulating BPIFB4 protein protected against carotid stenosis in human cohorts [12]. Contrariwise, BPIFB4 is reportedly downregulated in the heart of patients with end-stage ischemic heart failure [11].

Importantly, we have provided substantial evidence for the possibility of transferring the healthy phenotype conferred by *LAV-BPIFB4* to cardiovascular animal models, suggesting that temporary expression of an evolutionary successful human gene can halt and even reverse age-related damage. *LAV-BPIFB4* gene therapy in mice demonstrated anti-atherosclerotic [12], anti-hypertensive, pro-angiogenic [8, 11], and neuroprotective activities [13, 14]. Moreover, it improved frailty indices [15] and diabetic and age-related cardiomyopathies [11, 16], and rejuvenated the elderly vasculature [11, 17]. In addition, replicating the preserved immune function of centenarians [18], the LAV-BPIFB4 protein encouraged immunomodulatory responses by human myeloid cells [19, 20].

In the present study, we assessed the association of BPIFB4 expression and CAD severity in a cohort of patients with acute myocardial infarction (MI). We also conducted a preclinical study of *LAV-BPIFB4* gene therapy in a murine model of MI. Finally, we tested the effect of the LAV-BPIFB4 protein on human cardiomyocytes and cardiac fibroblasts.

## Results

### Low blood levels of BPIFB4 are associated with three-vessel CAD in patients with acute MI

We first investigated if the expression levels of BPIFB4 are inversely correlated with the severity/extension of CAD. Within a cohort of 492 patients with acute MI who entered the study, angiography data were available for 490 subjects. Of these patients, 181 (37%) were diagnosed to have evidence of three-vessel CAD, the most severe form of coronary artery atherosclerosis (Table 1). Compared with the remaining, this subgroup was slightly older (median value = 71 vs. 67 years, *p* = 0.0027), comprised more male subjects (78% vs. 62%, *p* = 0.0001), had more risk factors and comorbidities, including anemia, chronic kidney disease, diabetes, and peripheral artery disease, and scored worse in the Killip and GRACE classifications (*p* < 0.01 for all comparisons). Moreover, as expected, three-vessel CAD patients had more marked LV systolic dysfunction as assessed by echocardiography, were taking more drugs, such as ACE inhibitors, nitrates, insulin, aspirin, and statins, more frequently underwent coronary artery bypass graft surgery as a method of revascularization, and more likely experienced a previous MI (*p* < 0.05).

**Table 1:** Distribution of variables in the whole cohort of myocardial infarction patients and in subgroups classified according to the three-vessel CAD dependent variable.

				All^a^		Three-vessel CAD = No		Three-vessel CAD = Yes		
				(*n* = 492)		(*n* = 309)		(*n* = 181)		
Variable	Obs	F-miss (%)	Value	*N*	Distribution	*N*	Distribution	*N*	Distribution	*p*
Sex	492	0.00	Females	158	32.11%	118	38.19%	39	21.55%	0.0001
			Males	334	67.89%	191	61.81%	142	78.45%	
Age (years)	492	0.00		492	68 (59, 76)	309	67 (57, 76)	181	71 (63, 76)	0.0027
BMI (kg/m^2^)	490	0.41		490	26.23 (23.94, 29.41)	307	26.42 (23.88, 29.69)	181	26.15 (24.02, 29.3)	0.7113
CAD duration (months)	464	5.69		464	0 (0, 6.1)	292	0 (0, 0.34)	170	0 (0, 34.14)	0.0064
*RISK FACTORS AND COMORBIDITIES*										
Anemia	490	0.00	No	361	73.67%	244	79.48%	116	64.09%	0.0002
			Yes	129	26.33%	63	20.52%	65	35.91%	
Chronic kidney disease	490	0.00	No	444	90.61%	290	94.16%	153	84.53%	0.0004
			Yes	46	9.39%	18	5.84%	28	15.47%	
Diabetes	491	0.00	No	364	74.13%	241	78.25%	122	67.40%	0.0081
			Yes	127	25.87%	67	21.75%	59	32.60%	
Dyslipidemia	490	0.00	No	200	40.82%	135	43.83%	65	35.91%	0.0854
			Yes	290	59.18%	173	56.17%	116	64.09%	
Hypertension	490	0.00	No	138	28.16%	94	30.52%	44	24.31%	0.1407
			Yes	352	71.84%	214	69.48%	137	75.69%	
Peripheral artery disease	490	0.00	No	453	92.45%	296	96.10%	156	86.19%	0.0001
			Yes	37	7.55%	12	3.90%	25	13.81%	
*MI CLASSIFICATION AND RISK INDEXES*										
Type	489	0.01	NSTEMI	190	38.85%	110	35.83%	79	43.89%	0.0782
			STEMI	299	61.15%	197	64.17%	101	56.11%	
Previous MI	491	0.00	No	403	82.08%	264	85.71%	137	75.69%	0.0053
			Yes	88	17.92%	44	14.29%	44	24.31%	
Family History for CAD	489	0.01	No	375	76.69%	244	79.22%	130	72.22%	0.0779
			Yes	114	23.31%	64	20.78%	50	27.78%	
NYHA class	477	0.03	1	421	88.26%	270	89.40%	150	86.21%	0.5785
			2	40	8.39%	23	7.62%	17	9.77%	
			3/4	16	3.35%	9	2.98%	7	4.02%	
Killip classification > 1	491	0.00	No	372	75.76%	246	79.87%	125	69.06%	0.0070
			Yes	119	24.24%	62	20.13%	56	30.94%	
GRACE score at 6 months	489	0.61		489	119 (97, 140)	306	112 (93, 136.75)	181	129 (109, 150)	<0.0001
*MAIN ECHOCARDIOGRAPHY INDEXES*										
EDV (cm^2^)	464	5.69		464	47.2 (39.61, 57.65)	291	46.14 (38.71, 55.38)	171	50.08 (41.55, 61.71)	0.0024
ESV (cm^2^)	456	7.32		456	22.1 (16.71, 29.47)	286	20.75 (15.98, 28.4)	168	23.84 (17.87, 34.02)	0.0017
LV mass (g)	358	27.24		358	213 (174, 255.75)	225	208 (164, 247)	131	220 (187.5, 271)	0.0149
LVEF (%)	476	3.25		476	53 (45, 59)	297	55 (46, 60)	177	51 (42, 57)	0.0019
E/A	415	15.65		415	0.86 (0.67, 1.23)	270	0.86 (0.68, 1.22)	144	0.86 (0.67, 1.25)	0.6519
*LABORATORY TESTS*										
BPIFB4 (pg/ml)	492	0.00		492	69.13 (29.28, 153.88)	309	76.37 (34.52, 159.34)	181	56.87 (23.34, 118.91)	0.0077
Ln BPIFB4 (pg/ml)	492	0.00		492	4.24 (3.38, 5.04)	309	4.34 (3.54, 5.07)	181	4.04 (3.15, 4.78)	0.0077
BNP (pg/ml)	236	52.03		236	51.92 (21.25, 84.27)	145	54.66 (29.16, 93.84)	90	48.6 (13.16, 70.2)	0.0273
HbA1C (%)	334	32.11		334	6 (5.7, 6.6)	195	5.9 (5.6, 6.5)	138	6.1 (5.8, 6.77)	0.0078
Hs CRP (mg/dl)	474	3.66		474	4.4 (1.6, 11.38)	295	4 (1.4, 9.9)	177	4.8 (1.8, 14.9)	0.0545
MDRD (ml/min)	480	2.44		480	62.85 (50.16, 76.78)	300	66.12 (53.58, 80.03)	179	57.61 (45.87, 70.94)	<0.0001
Tnl max (ng/ml)	481	2.24		481	13.79 (2.99, 53)	304	14 (2.55, 53.25)	175	12.5 (3.55, 51.06)	0.8491
*TREATMENT*										
Treatment	480	0.02	PCI	343	71.46%	220	73.58%	123	68.33%	<0.0001
			CABG	53	11.04%	12	4.01%	41	22.78%	
			Medical Therapy	84	17.50%	67	22.41%	16	8.89%	
*TYPE OF DRUG*										
ACE Inhibitors	484	0.02	No	258	53.31%	179	58.50%	78	44.07%	0.0022
			Yes	226	46.69%	127	41.50%	99	55.93%	
Beta blockers	485	0.01	No	349	71.96%	229	74.59%	120	67.80%	0.1083
			Yes	136	28.04%	78	25.41%	57	32.20%	
Calcium channel blockers	485	0.01	No	400	82.47%	257	83.71%	143	80.79%	0.4136
			Yes	85	17.53%	50	16.29%	34	19.21%	
Nitrates	484	0.02	No	426	88.02%	282	92.16%	143	80.79%	0.0002
			Yes	58	11.98%	24	7.84%	34	19.21%	
Thiazides	485	0.01	No	438	90.31%	277	90.23%	160	90.40%	0.9522
			Yes	47	9.69%	30	9.77%	17	9.60%	
Loop diuretics	485	0.01	No	441	90.93%	283	92.18%	157	88.70%	0.1994
			Yes	44	9.07%	24	7.82%	20	11.30%	
Aldosterone antagonists	485	0.01	No	471	97.11%	296	96.42%	174	98.31%	0.2326
			Yes	14	2.89%	11	3.58%	3	1.69%	
Insulin	485	0.01	No	458	94.43%	295	96.09%	162	91.53%	0.0350
			Yes	27	5.57%	12	3.91%	15	8.47%	
Oral antidiabetic drugs	486	0.01	No	405	83.33%	263	85.67%	141	79.21%	0.0662
			Yes	81	16.67%	44	14.33%	37	20.79%	
Statins	485	0.01	No	358	73.81%	237	77.20%	121	68.36%	0.0329
			Yes	127	26.19%	70	22.80%	56	31.64%	
Allopurinol	485	0.01	No	464	95.67%	297	96.74%	166	93.79%	0.1240
			Yes	21	4.33%	10	3.26%	11	6.21%	
Aspirin	485	0.01	No	338	69.69%	228	74.27%	110	62.15%	0.0051
			Yes	147	30.31%	79	25.73%	67	37.85%	
Anti-platelet drugs	485	0.01	No	443	91.34%	286	93.16%	156	88.14%	0.0586
			Yes	42	8.66%	21	6.84%	21	11.86%	
Heparin	485	0.01	No	466	96.08%	296	96.42%	169	95.48%	0.6093
			Yes	19	3.92%	11	3.58%	8	4.52%	

*Variable* analyzed variable, *Obs.* non-missing observations, *F-miss (%)* frequency (%) of missing values, *Value* value that each categorical variable assumes, *N* number of observations, *Distribution* relative frequency of categorical variables’ values in the whole cohort and in patients with and without three-vessel CAD or median (25th, 75th percentiles) of numeric variables distribution; *p* value *p* value (Wilcoxon rank-sum test, Pearson chi-square test or Fisher’s exact test for independence based on variables’ distribution) comparing variables’ distribution between patients with and without three-vessel CAD.

^a^The number of patients affected by three vessel CAD and the number of patients not affected by three vessel CAD do not sum to the total number of patients due to the presence of two patients with unknown three vessel CAD status.

Three-vessel CAD patients were further characterized by lower brain natriuretic peptide (BNP) and modification of diet in renal disease estimated GFR (MDRD) while having higher HbA1c levels (*p* < 0.05). Moreover, they had significantly lower levels of the natural logarithm (Ln) transformed BPIFB4 (*p* = 0.0077). Importantly, logistic regression showed an inverse relationship between Ln BPIFB4 levels and three-vessel CAD both in an unadjusted model (Odds Ratio [OR] = 0.83, 95% Confidence Interval [CI] = 0.72–0.96, *p* = 0.0107) and in a model adjusted for dyslipidemia, nitrate therapy, GRACE and previous MI score performed on data from 481 patients with complete information for the analyzed variables (OR = 0.81, 95% CI = 0.70–0.94, *p* = 0.0054). These variables were included in the multivariate model since they represented potential confounders, showing evidence of association both to Ln BPIFB4 levels (*p* < 0.10) (Table 2) and three-vessel CAD (*p* < 0.10) (Table 1). Of note, when all variables reported in Table 2 were included in multivariate logistic regression as potential confounders, the association between Ln BPIFB4 levels and three-vessel CAD remained statistically significant, further confirming the robustness of the finding (n. patients with complete information for the analyzed variables = 420, OR = 0.77, 95% CI = 0.63–0.92, *p* = 0.0053).

**Table 2:** Correlation between log-transformed (Ln) BPIFB4 levels and potentially informative variables in myocardial infarction patients.

				Ln BPIFB4	
Variable	Obs	Value	*N*	Distribution/r	*P*
Three vessel CAD	490	No	309	4.34 (3.54, 5.07)	0.0077
		Yes	181	4.04 (3.15, 4.78)	
*ANTHROPOMETRIC/DEMOGRAPHIC DATA*					
Age	492		492	0.06 [−0.03, 0.15]	0.1904
Sex	492	No	158	4.21 (3.25, 5.03)	0.7583
		Yes	334	4.24 (3.43, 5.04)	
BMI (Kg/m^2^)	490		490	−0.02 [−0.11, 0.07]	0.5930
*RISK FACTORS AND COMORBIDITIES*					
Anemia	490	No	361	4.24 (3.42, 5.06)	0.6128
		Yes	129	4.2 (3.31, 4.94)	
Chronic kidney disease	490	No	444	4.22 (3.38, 5.05)	0.7963
		Yes	46	4.39 (3.34, 5)	
Diabetes	491	No	364	4.24 (3.29, 5.06)	0.7410
		Yes	127	4.2 (3.59, 4.92)	
Dyslipidemia	490	No	200	4.46 (3.59, 5.09)	0.0127
		Yes	290	4.11 (3.23, 5)	
Hypertension	490	No	138	4.31 (3.36, 5.02)	0.8936
		Yes	352	4.18 (3.38, 5.04)	
Peripheral artery disease	490	No	453	4.23 (3.36, 5.03)	0.8425
		Yes	37	4.24 (3.59, 5.08)	
*MI CLASSIFICATION AND RISK INDEXES*					
Type	489	NSTEMI	190	4.32 (3.4, 5.01)	0.7885
		STEMI	299	4.21 (3.37, 5.05)	
Previous MI	491	No	403	4.3 (3.45, 5.06)	0.0787
		Yes	88	3.88 (3.17, 4.76)	
Family History for CAD	489	No	375	4.26 (3.4, 5.06)	0.4446
		Yes	114	4.1 (3.36, 4.94)	
NYHA class	477	1	421	4.22 (3.42, 5.04)	0.9427
		2	40	4.23 (3.27, 4.9)	
		3/4	16	4.33 (2.98, 5)	
Killip classification > 1	491	No	372	4.2 (3.29, 5)	0.1318
		Yes	119	4.51 (3.58, 5.07)	
GRACE score at 6 months	489		489	0.1 [0.01, 0.19]	0.0283
*ECHOCARDIOGRAPHY AND LABORATORY TESTS*					
LV Ejection Fraction	476		476	−0.06 [−0.15, 0.03]	0.1921
HS CRP (mg/dL)	474		474	0.04 [−0.05, 0.13]	0.3564
MDRD (mL/min)	480		480	−0.03 [−0.12, 0.06]	0.4794
Tnl max (ng/mL)	481		481	−0.01 [−0.1, 0.08]	0.7540
*TREATMENT*					
Treatment	480	PCI	343	4.2 (3.38, 4.98)	0.1783
		CABG	53	4.31 (3.29, 5.09)	
		Medical Therapy	84	4.55 (3.59, 5.28)	
*ONGOING THERAPY*					
ACE Inhibitors	484	No	258	4.26 (3.37, 5.01)	0.9680
		Yes	226	4.14 (3.33, 5.06)	
Beta Blockers	485	No	349	4.25 (3.36, 5.01)	0.7551
		Yes	136	4.16 (3.43, 5.14)	
Ca2+ Channel Blockers	485	No	400	4.2 (3.41, 4.99)	0.5230
		Yes	85	4.48 (3.1, 5.24)	
Nitrates	484	No	426	4.27 (3.47, 5.06)	0.0150
		Yes	58	3.73 (3, 4.62)	
Thiazide	485	No	438	4.22 (3.28, 5)	0.0985
		Yes	47	4.26 (3.62, 5.32)	
Loop diuretics	485	No	441	4.24 (3.39, 5.06)	0.3526
		Yes	44	4.02 (3.06, 4.77)	
Aldosterone antagonists	485	No	471	4.23 (3.37, 5.04)	0.9499
		Yes	14	3.94 (3.26, 5.15)	
Insulin	485	No	458	4.23 (3.36, 5.04)	0.9910
		Yes	27	4.11 (3.48, 4.91)	
Oral antidiabetic drugs	486	No	405	4.22 (3.31, 5.03)	0.3911
		Yes	81	4.41 (3.66, 5.06)	
Statins	485	No	358	4.28 (3.37, 5.05)	0.3663
		Yes	127	4.01 (3.24, 5.01)	
Allopurinol	485	No	464	4.24 (3.37, 5.06)	0.4797
		Yes	21	4.01 (3.31, 4.75)	
Aspirin	485	No	338	4.27 (3.42, 5.06)	0.2030
		Yes	147	4.11 (3.24, 5.01)	
Anti-platelet drugs	485	No	443	4.25 (3.41, 5.05)	0.1649
		Yes	42	3.99 (3.1, 4.64)	
Heparin	485	No	466	4.24 (3.38, 5.06)	0.2325
		Yes	19	3.97 (3.09, 4.44)	

Total cohort included 492 patients, but data regarding the presence of three-vessel CAD was missing in 2 patients.

*Variable* analyzed variable, *Obs*. number of non-missing observations, *Value* value that each categorical/ordinal variable assumes, *N* number of observations, *Distribution/r* median (25th, 75th percentile) of Ln BPIFB4 distribution by categorical variables or Spearman correlation coefficient *r* [95% Confidence Interval] quantifying the degree of correlation between Ln BPIFB4 and numeric continuous/discrete variables, *p value p* value from Wilcoxon rank-sum test for independent samples comparing Ln BPIFB4 distribution between variables’ levels, from the Kruskal–Wallis test comparing Ln BPIFB4 distribution among variables’ levels, or from the Spearman correlation test. The reported variables had a frequency of missing values < 5% and were considered as potential confounders to be included in multivariate models (variables CAD duration, EDV, ESV, LV mass, E/A, BNP, HbA1C are excluded from the table for missing data fraction > 5%).

### LAV-BPIFB4 gene therapy protects the heart from ischemia

We next performed preclinical studies of *LAV-BPIFB4* gene therapy in a murine model. We have previously shown that a single *LAV-BPIFB4* injection produced a long-term expression of the protein in the murine heart [11, 16]. Moreover, new data on C57BL/6 mice indicate significantly increased levels of BPIFB4 in peripheral circulation and improved vascular reactivity as soon as 4 days after gene therapy (Puca, unpublished data 2021).

**Fig. 1:**
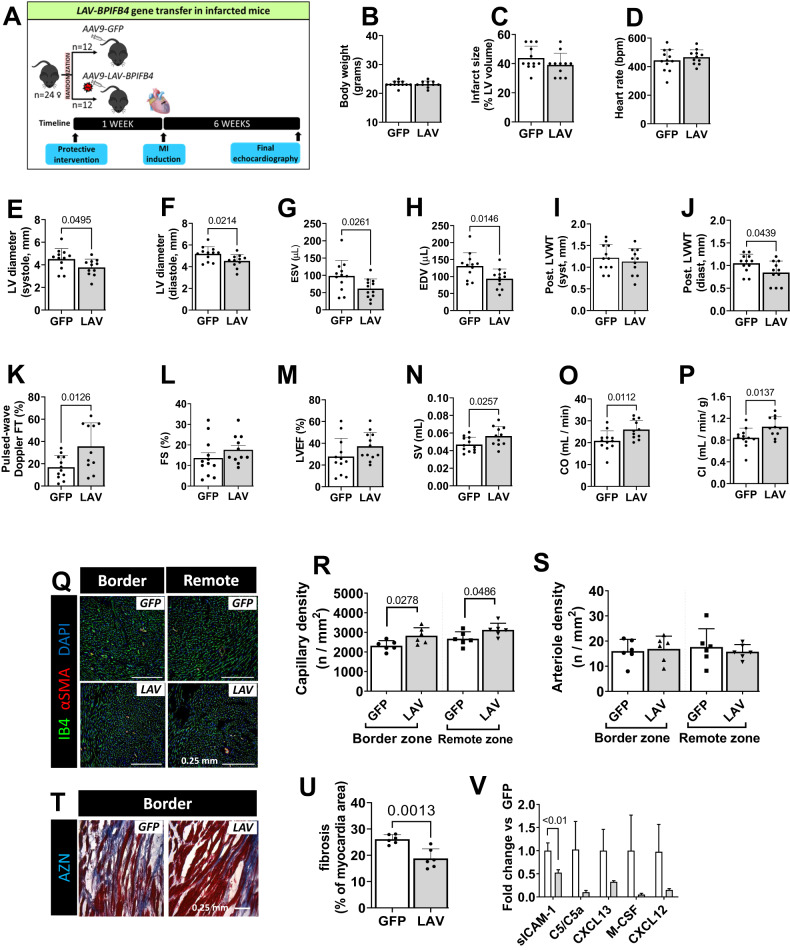
A single systemic injection of AAV- *AV-BPIFB4* attenuates the cardiovascular damage of acute MI in mice. **A** Schematic of the experimental protocol with a total of 24 female mice randomized (1:1 ratio) to the 2 arms of the study. **B** Body weight at the end of the study. **C** Infarct size calculated at histology. **D–P** Echocardiography data were assessed before termination to measure heart rate (**D**), left ventricular diameters and volumes in systole and diastole (**E–H**), posterior left ventricular wall thickness (LVWT) in diastole and systole (**I, J**), Pulsed-wave Doppler FT (**K**), fractional shortening (FS) (**L**), ejection fraction (LVEF) (**M**), stroke volume (SV) (**N**), cardiac output (CO) (**O**), and cardiac index (CI) (**P**). **Q–S** Vascular density at the level of the peri-infarct border zone and remote zone. **Q** Representative fluorescent microscopy images showing endothelial cells and vascular smooth muscle cells labelled by Isolectin B4 (IB4, green) and α-smooth muscle actin (αSMA, red), respectively. **R**, **S** Bar graphs showing capillary (**R**), and arteriole density (**S**)**. T**, **U** Fibrosis was assessed by Azan Mallory staining (blue). Representative microscopy images (**T**) and bar graphs showing the values in the two groups (**U**). **V** Results of an array assessing the levels of circulating inflammatory factors. Data were analyzed using parametric tests. Data are presented as individual values and standard deviation.

Based on these data, we designed a preventive intervention where female mice were IV injected with an AAV vector, carrying *LAV-BPIFB4* or *GFP*, 1 week before induction of MI (Fig. 1A). The two groups were similar regarding body weight, infarct size, and heart rate (HR) (Fig. 1B–D). At the end of the follow-up (6 weeks post-MI), we found that we found that, compared with controls, *LAV-BPIFB4*-treated mice had lower LV systolic and diastolic diameters (−16% and −13%, respectively) and volumes (−38% and −28%, respectively) (Fig. 1E–H). The LV wall thickness was reduced in diastole (−20%) but not in systole (Fig. 1I, J). Moreover, as shown in *Figure K-P*, the *LAV*-treated group showed improved indexes of LV function, including increases in pulsed-wave Doppler FT (2.0-fold), stroke volume (1.2-fold), cardiac output (1.3-fold), and cardiac index (1.2-fold). However, the difference in fractional shortening and ejection fraction did not reach statistical significance. Histological analyses demonstrated a higher capillary density in the myocardium of the *LAV*-*BPIFB4* treated group (1.2-fold vs. *GFP*) whereas the arteriole density was similar (Fig. 1Q–S). The *LAV*-*BPIFB4-*treated group showed a lower extension of fibrosis in the peri-infarct border zone (−28% vs. *GFP*) (Fig. 1T, U). Moreover, a cytokine array demonstrated that *LAV-BPIFB4* induced a global reduction in the circulating levels of inflammatory cytokines which reached statistical significance for soluble intercellular adhesion molecule-1 (sICAM-1) (Fig. 1V and Supplementary Fig. 1A).

### LAV-BPIFB4 exerts inotropic and chronotropic effects on cardiomyocytes

**Fig. 2:**
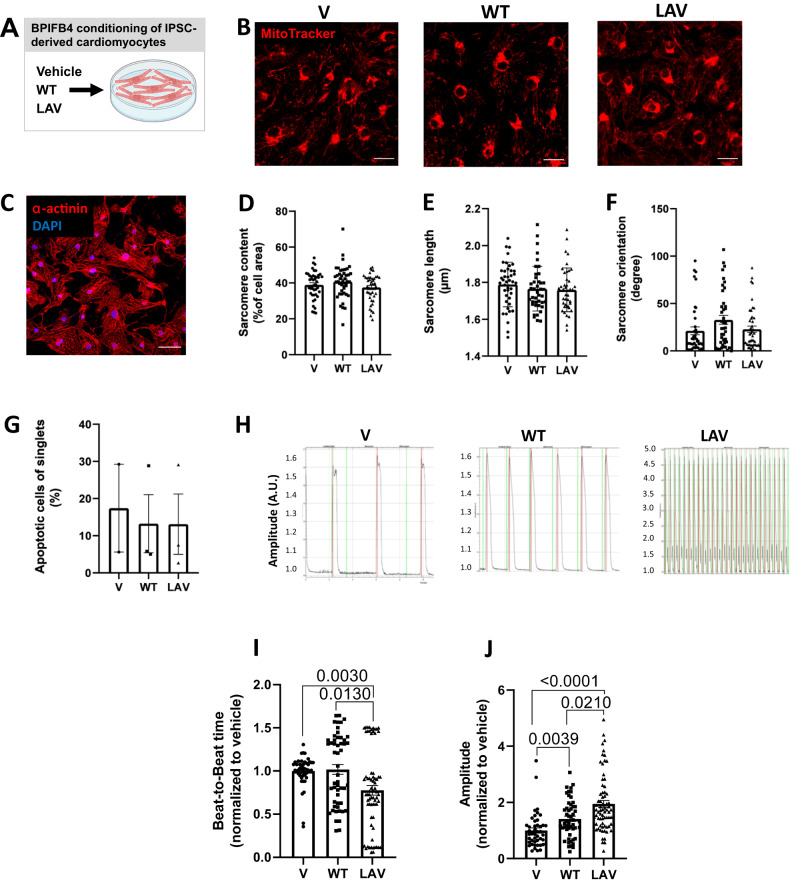
LAV-BPIFB4 exerts chronotropic and inotropic effects on isolated cardiomyocytes. **A** Cardiomyocytes were derived from human iPSCs and exposed to BPIFB4 recombinant proteins (WT and LAV) or vehicle (V) in 2–4 independent rounds of cardiac differentiation. Bar scale, 20 μm. **B** Illustrative images of MitoTracker staining. **C–F** Data of sarcomere dimensions. Typical staining of α-actinin (red). Bar scale, 50 μm (**C**). Bar graph showing sarcomere content (**D**), length (**E**), and orientation (**F**). **G** Effect of LAV-BPIFB4 on cell apoptosis. **H–J** Functional data: Representative traces (**H**), and bar graphs showing time between single beats (**H, I**) and the amplitude of contraction (**J**). *n* = 20–80. Data were analyzed using non-parametric tests.

We next asked whether supplementation of BPIFB4 protein may impact cardiomyocyte function. To this aim, we exposed iPSC-derived cardiomyocytes to BPIFB4 isoforms or vehicle (Fig. 2A). Like adult counterparts, iPSC-derived cardiomyocytes were rich in mitochondria, recognized by MitoTracker Red staining. WT-BPIFB4 and LAV-BPIFB4 proteins did not affect mitochondria (Fig. 2B) or sarcomere content (Fig. 2C, D). Also, no differences were detected in sarcomere length and filament orientation (Fig. 2E, F) [21]. Likewise, no effect on cell apoptosis was observed following treatment with BPIFB4 isoforms (Fig. 2G). The expression of BPIFB4 was identified in the cell cytoplasm (Supplementary Fig. 2). Looking at functional indexes, we found that only LAV-BPIFB4 significantly decreased the average beat-to-beat time, reflecting higher beating frequencies (Fig. 2H, I). Similarly, the contraction amplitude, which corresponds to force development, was significantly increased by both isoforms, yet, with a remarkably higher effect of LAV-BPIFB4 (Fig. 2J). These data indicate that LAV-BPIFB4 exerts chronotropic and inotropic effects on isolated cardiomyocytes.

### LAV-BPIFB4 antagonizes TGF-β1 induction of fibrotic markers

**Fig. 3:**
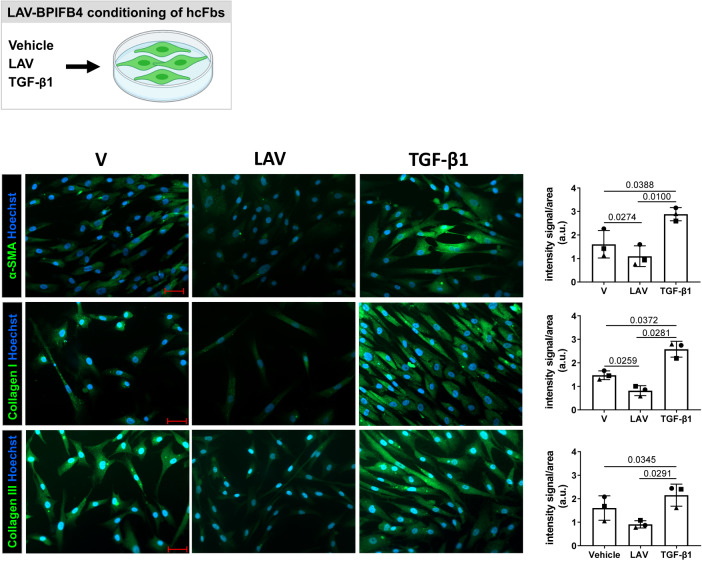
LAV-BPIFB4 reduces the cardiac fibroblast pro-fibrotic phenotype. HcFbs were stimulated with the recombinant LAV-BPIFB4 protein or Vehicle. TGF-β1 (10 ng/ml) was used as positive control. In the left panel, representative images of α-SMA, Collagen I and Collagen III stained in green; nuclei were stained with Hoechst (blue). Bar scale, 50 μm. In the right panel, quantification of α-SMA, Collagen I and Collagen III expression. Symbols represent subjects (circle, 74-year-old donor; square, 50-year-old donor and triangle, 34-year-old donor). Bar graphs represent mean ± SD (*n* = 3). Data were analyzed using parametric-tests.

Notably, in vitro passage of cardiac fibroblasts in the absence of TGF-β1 stimulation is sufficient to increase the expression of canonical TGF-β1 signaling effectors and induce the myofibroblast phenotype [22]. Thus, we evaluated the effect of recombinant LAV-BPIFB4 protein on the spontaneous pro-fibrotic activity of hcFbs [23, 24]. Cell lines from three female donors (Supplementary Table 1) were exposed to recombinant LAV-BPIFB4 protein, vehicle, or TGF-β1, the latter as an inducer of fibroblast activation. As expected, TGF-β1 increased the cellular expression of α-SMA, Collagen I, and Collagen III proteins (Fig. 3). Interestingly, LAV-BPIFB4 supplementation significantly reduced the fibrotic markers α-SMA and Collagen I compared with the vehicle, whereas the down-modulation in the protein level of Collagen III did not reach statistical significance (Fig. 3). Next, we further explored the impact of LAV-BPIFB4 on TGF-β1-induced pro-fibrotic response by exposing hcFbs to the combined LAV-BPIFB4 and TGF-β1 supplementation. LAV-BPIFB4 attenuated the TGF-β1-induced increase in pro-fibrotic proteins, with the statistical significance being reached for Collagen I (Supplementary Fig. 3). BPIFB4 localized mainly in the cytoplasmic compartment in both control and treated cells (Supplementary Fig. 4).

## Discussion

The present study provides compelling evidence for the protective role of BPIFB4 and its longevity-associated variant against heart disease. We showed an inverse association between BPIFB4 and three-vessel CAD severity, a protective effect of *LAV-BPIFB4* gene delivery in a model of MI, and a positive impact of LAV-BPIFB4 protein on human cardiomyocytes and cardiac fibroblasts.

### Downregulation of BPIFB4 marks poor cardiovascular outcomes in older people

The human *BPIFB4* gene encodes a secreted protein, initially found to be expressed in salivary glands and olfactory epithelia to confer microbial resistance. It belongs to a class of olfactory proteins and cognate receptors that regulate proteostasis and longevity, possibly through brain-to-gut signalling [25–28]. Their downregulation, along with olfactory dysfunction, reportedly predicts degenerative disease and death in the elderly [29, 30].

Our previous studies showed that carriers of the *LAV-BPIFB4* polymorphism have high blood levels of the encoded protein and enjoy prolonged healthy lifespans [8], whereas a rare variant (RV; allele frequency, 4%) was associated with arterial hypertension and endothelial dysfunction [31]. Moreover, recent clinical studies reported the inverse correlation between the LAV-BPIFB4 genotype, the pathological intima-media thickness [12], and the scarcity and dysfunction of pericytes in the heart of patients with ischemic heart failure [11]. In accordance, low BPIFB4 mRNA transcript and protein were previously reported in the epicardial adipose tissue of CAD patients [32] and in elderly failing human hearts [11]. Here, we report new findings from a human cohort indicating that the downregulation of BPIFB4 in peripheral blood is associated with multiple vessels CAD in a multivariate model.

### LAV-BPIFB4 gene therapy protects the infarcted murine heart

Treatment with LAV-BPIFB4 improved cardiac index (primary endpoint), microvascular density, and interstitial fibrosis (secondary endpoints). A closer analysis of echocardiographic data indicates the LAV-BPIFB4-treated mice had reduced volumetric dimensions and improved systolic function, which is in keeping with the in vitro data showing improved contractility of LAV-BPIFB4-treated cardiomyocytes (*vide infra*). These results agree with previous results in diabetic and aging mice [11, 16]. The anti-fibrotic effects exerted by LAV-BPIFB4 may be in part reconducted to its capability to modulate the circulating soluble cytokine levels, especially ICAM-1, a crucial driver of proinflammatory leukocyte infiltration and fibrosis whose plasma levels are predictive for MI [33, 34].

The limitations of the MI study are the lack of a sham surgery control group and the use of female mice only. There is a controversy on the ethical justification for adding a sham surgery comparator when performing a study testing efficacy of an active drug vs. placebo in MI mice. We used female mice because they represent the lesser severe model suitable to verify the experimental hypothesis. Additional studies are needed to confirm the benefit in male mice.

### LAV-BPIFB4 exerts direct effects on human cardiomyocytes and cardiac fibroblasts

Interestingly, LAV-BPIFB4 induced a chronotropic effect and potently increased the amplitude of the contraction, the latter effect being also observed, although to a lesser degree, after WT-BPIFB4 stimulation. We previously showed that *LAV-BPIFB4* gene therapy induced an up-regulation of the cardiac MyHC-α, a contractile protein that is reduced in diabetic and failing hearts [16]. Moreover, LAV-BPIFB4 can increase calcium mobilization through the phosphorylation and translocation of protein kinase C alpha (PKCα) [35]. Within the cytoplasmatic compartment of vascular cells, BPIFB4 interacted with a subset of proteins (e.g., 14-3-3 and HSP90), activating NO and PKCα signaling [8]. Similar mechanisms are likely responsible for the functional improvements observed in isolated cardiac cells.

In accordance with the anti-fibrotic effects observed in vivo, LAV-BPIFB4 supplementation to hcFbs decreased the protein expression of the main fibrotic markers either during the spontaneous or the TGF-β1-stimulated fibrogenesis. Quiescent fibroblasts can differentiate into myofibroblasts, as identified by de novo expression of αSMA and secretion of extracellular matrix proteins [36]. Activated cardiac fibroblasts represent myocardial fibrosis’s primary driver of systolic and diastolic dysfunction in cardiac disease [36, 37]. By positively modulating the cardiomyocyte and cardiac fibroblast functions, LAV-BPIFB4 may preserve the homeostasis of the myocardial environment and protect from the adverse fibrotic remodeling of the infarcted heart.

### Conclusions

In this study, we show that the levels of BPIFB4 expression contribute to the heart’s functional state during ischemia. While low BPIFB4 characterizes severe CAD in patients, forced expression of the longevity variant revitalized the function and vascularization of infarcted hearts in female mice. In compliance with the 3 R guidelines and the ethical licence covering this study, male mice were not investigated as they have higher mortality rates and worse outcomes after an MI. Moreover, we have already shown that sex does not influence the benefit of LAV-BPIFB4 therapy on the heart [11]. Additional efficacy/safety studies toward regulatory approval of the longevity gene/protein are necessary to determine if this new technology can become a viable treatment for myocardial infarction.

## Materials and methods

An extended Methods version is reported as Online Supplementary Material. The data underlying this article will be shared upon reasonable request.

### Clinical study

#### Association of BPIFB4 expression and three-vessel CAD in a cohort of myocardial infarction patients

The extension of CAD was assessed by angiography in a consecutive series of 492 patients hospitalized for acute myocardial infarction (MI) at the University Hospital of Trieste from May 2014 to March 2017. The study was approved by the Local Ethics Committee (protocol n. 67/2015).

Clinical data are reported in Table 1 Inclusion criteria were age >18 years, MI with clinical onset in the previous 24 h, and written informed consent for study participation. Exclusion criteria were active malignancy with a life expectancy <12 months and inability to understand the nature and purpose of the study. The peripheral blood levels of BPIFB4 and brain natriuretic peptide (BNP) were determined using ELISA kits (Cusabio and RayBiotech, Norcross, USA, respectively).

### Gene therapy studies in mice

Experimental procedures complied with the EU Directive 2010/63/EU and principles stated in the Guide for the Care and Use of Laboratory Animals (Institute of Laboratory Animal Resources, 1996). Methods and reagents are shown in *Supplementary Materials* and Supplementary Table 2.

#### Preventive *LAV-BPIFB4* gene therapy in mice with MI

##### Objective

The study, conducted at the University of Rouen, aimed to assess the efficacy of *AAV-LAV-BPIFB4* gene therapy in preventing cardiac dysfunction caused by an MI.

##### Endpoints

Cardiac index (primary endpoint) and vascular density (secondary endpoint).

##### Protocol

The animal protocol was approved by Haute-Normandie Ethics Board (authorization no. 01307.01). Two-month-old female C57Bl/6J mice (Janvier Labs, Le Genest-Saint-Isle, France) were randomized to receive 100 μL of 1 × 10^12^ GC/mL *AAV9-LAV-BPIFB4 or* control *AAV9-GFP* (ratio of sample size = 1:1) through the tail vein (*n* = 12/treatment group). One week later, animals underwent permanent ligation of the left anterior descending (LAD) coronary artery under isoflurane anesthesia. Mice were examined every day during the first week post-MI and then weekly. Six weeks after MI (end of the study), cardiac function was assessed using echocardiography (Vevo 3100, FUJIFILM VisualSonics, Toronto, Canada). After imaging, anesthetized animals were sacrificed by blood sampling. Hearts were snap-frozen and stored at −80 °C for immunohistological analyses.

### Statistical analyses

An expanded version of statistics can be found in *Supplementary Materials* The comparison of numeric variables distribution between binary variables was performed by the Student’s *t* test or with the equivalent non-parametric test. When appropriate, one-way ANOVA (followed by Tukey’s multiple comparisons tests) or Kruskal–Wallis tests (followed by Dunn’s multiple comparison tests) were employed. Comparison among groups with two independent variables was performed employing two-way ANOVA followed by Sidak’s multiple comparison test. Analyses were conducted with GraphPad Prism 8.0 for MacOS or 8.4.3 for Win.

In clinical study, the BPIFB4 values were transformed due to their extremely right skewed distribution using natural logarithm (Ln) to make BPIFB4 distribution more symmetric. Logistic regression was used to test for association between BPIFB4 values and the occurrence of three-vessel CAD. The significance level has been set to α = 0.05. Statistical analyses have been performed by the R software environment for statistical computing and graphics version 4.0.5 (www.r-project.org).

## Supplementary information

Supplementary data

Supplementary Figure 1

Supplementary Figure 2

Supplementary Figure 3

Supplementary Figure 4

Supplementary Table 1

Supplementary Table 2

## Data Availability

All data generated or analyzed during this study are included in this published paper and its Supplementary Information files. Additional data are available from the corresponding author on reasonable request.
