# Supplementary material for: BPIFB4 and its longevity-associated haplotype protect from cardiac ischemia in humans and mice
Source: Cell Death Dis. 2023 Aug 15;14(8):523. doi: 10.1038/s41419-023-06011-8 (PMC10427721; doi:10.1038/s41419-023-06011-8)
Supplement: Supplementary file 1 — Supplementary data [file 41419_2023_6011_MOESM1_ESM.docx]

**EXPANDED ONLINE METHODS**

**Association of BPIFB4 expression and three-vessel CAD in a cohort of myocardial infarction patients.**

The extension of CAD was assessed by angiography in a consecutive series of 492 patients hospitalized for acute myocardial infarction (MI) at the University Hospital of Trieste from May 2014 to March 2017. The study was approved by the Local Ethics Committee (protocol n. 67/2015). The informed consent was obtained from all subjects.

Clinical data are reported in **Table 1** Inclusion criteria were age > 18 years, MI with clinical onset in the previous 24h, and written informed consent for study participation. Exclusion criteria were active malignancy with a life expectancy < 12 months and inability to understand the nature and purpose of the study. The peripheral blood levels of BPIFB4 and brain natriuretic peptide (BNP) were determined using ELISA kits (Cusabio and RayBiotech, Norcross, USA, respectively).

**Production of *Bpifb4*** **vectors and recombinant Bpifb4** **proteins**

The adeno-associated vector (AAV) was produced as described previously [1]. For each viral preparation, physical titers (GC/mL) were determined through dot-blot analysis and polymerase chain reaction quantification using TaqMan20 (Applied Biosystems, Carlsbad, USA).

Recombinant BPIFB4-His proteins expression and purification were previously described [2]. Protein samples purified from Hek-293 transfected with the empty vector was used as a negative control and referred to as vehicle. The protein concentration was determined using a Qubit fluorometer (Thermo Fisher Scientific).

***LAV-BPIFB4* gene transfer in mice with acute myocardial infarction**

Experimental procedures were compliant with the EU Directive 2010/63/EU and principles stated in the Guide for the Care and Use of Laboratory Animals (Institute of Laboratory Animal Resources, 1996). The protocols detailed below were prepared with support from the Experimental Design Assistant, a free resource from the National Centre for Replacement, Refinement, and Reduction of Animals in Research (<https://eda.nc3rs.org.uk/>), under French National Legislation. Mice were housed in an enriched environment within a bio-secure unit under a 12 h light/dark cycle, fed with EURodent Diet (5LF5, LabDiet, Durham, UK) and given drinking water *ad libitum*. The GraphPad software (https://www.graphpad.com/quickcalcs/randomize1/) was used to randomly assign subjects to treatment groups, which were coded to allow a blind assessment of the data.

*Objective:* The study, conducted at the University of Rouen (France), aimed to assess the efficacy of *AAV9-LAV-BPIFB4* gene therapy in preventing cardiac dysfunction caused by an MI. *Protocol:* Two-month-old female C57Bl/6J mice (Janvier Laboratories, France) were randomized to receive 100 μL of 1 × 10^12^ GC/mL *AAV9-LAV-BPIFB4 or AAV9-GFP* (ratio of sample size = 1:1) through the tail vein. One week later, animals underwent permanent ligation of the left anterior descending (LAD) coronary artery (n=12/treatment group) under isoflurane anesthesia. Mice were examined every day during the first week after the MI induction and then weekly for the remaining follow-up period. At 6 weeks after the MI induction (end of the study), At 6 weeks after the MI induction, mice were re-anesthetized (isoflurane 2% for induction, followed by 0.5–2% as appropriate to maintain heart rate close to 450 bpm) and assessed using echocardiography (Vevo 3100, FUJIFILM VisualSonics, Toronto, Canada). Then, mice were terminated under general anesthesia by blood sampling followed by tissue harvesting. The hearts were snap-frozen and stored at -80^o^C for subsequent immunohistological analyses as described previously [3,4]. *Endpoints:* Cardiac index (primary endpoint) and vascular density (secondary endpoint). *Null hypothesis:* The endpoints are not affected by the experimental manipulation (*AAV9-LAV-BPIFB4*) to be tested. *Primary endpoint’s effect size:* We considered a difference in cardiac index of 0.20 between groups to be of biological relevance. *Group size:* A minimum of 10 animals per group was considered necessary with an SD of 0.15, a p=0.05, and a power of 0.8. *Secondary endpoint’s effect size:* We considered a difference in capillary density of 400 in the peri-infarct zone to be biologically relevant [3-6]. *Group size:* A minimum of 6 animals per group was considered to be necessary with an SD of 225, a p=0.05, and a power of 0.8. The effect sizes were based on previous experiments conducted in the murine MI model [3-6].

**Tissue collection and histological analysis**

Blood was obtained into EDTA directly from the heart through a 23 gauge cannula. The heart was then flushed with 1% EDTA in normal phosphate-buffered saline (PBS), weighed, and then cut into pieces; the top 2/3 section was drop-fixed in 4% PFA, and the lower third was separated into the right ventricle (RV) and left ventricle LV and flash frozen. All PFA samples were kept at +4^o^C for 18-24 h and then the PFA was replaced with PBS. The PFA-fixed cardiac tissues were cryoprotected using 30% sucrose for 24 h before being embedded in OCT. Histochemical and immunohistochemical studies were performed on 4 μm thick sections cut using a Thermo Fisher Scientific CryoStar NK50 cryostat unless otherwise stated.

**Histological analyses of mouse hearts**

Specific antibodies and procedures are listed in **Supplementary Table 2**. All the immunochemical procedures included tissue sections without primary antibodies as a technical internal control and suitable irrelevant IgG as negative controls. Images were acquired employing either a transmitted light microscope (Leica DMD 108, Leica, Wetzlar, Germany or Olympus BS40), a confocal microscope (Leica TCS-SP8), or an epifluorescence microscope (Leica DMI 6000B or Zeiss AxioObserver.Z1 microscope).

Cryosections were stained using hematoxylin and eosin (H&E) or elastic van Gieson (EVG) protocols using a Shandon Varistan 24-4 slide stainer (Thermo Fisher Scientific). Slides were mounted with DPX. Further 8 μm thick sections were stained for collagen using the Azan Mallory method (Heidenhain’s adaption of Mallory’s trichrome stain). Cardiac muscles were stained with anti-α-sarcomeric actin (1:200, mouse IgM isotype, Sigma-Aldrich), for 2 hours, at RT and Alexa Fluor 647-conjugated anti-mouse IgM (1:200, for 1 hour, at +20°C, Life Technologies, UK) was used as a secondary antibody. Slides were stained with 1:1000 (v/v) DAPI solution in 1xPBS and mounted with Fluoromount G for the imaging. Representative and quantitative images were taken using a Zeiss Observer.Z1 microscope set up on a fluorescent field light path with a 20x objective. Vascular density was measured by counting capillaries and arterioles in >10 fields (200 x magnification). Final data were expressed as the number of capillaries and arterioles per mm^2^ [7]. Morphometric analyses were carried out employing ImageJ software Analysis of PCs associated with the coronary microvasculature was carried out on 2 to 3 sections/sample stained with antibodies anti-PDGFRβ (1:50, overnight +4°C, identifying PCs) and α-sarcomeric actin (1:100, overnight at +4°C), and with isolectin-B4 (IB4, 1:200, overnight +4°C, identifying vessel ECs). Morphometric analyses were carried out employing ImageJ software.

**Cytokine array profiler**

Murine plasma samples were assessed for a parallel determination of the relative levels of 40 mouse cytokines using a proteome profiler array according to the manufacturer’s instructions (ARY006, R&D Systems, Minneapolis, USA). Briefly, the membranes retaining the capture antibodies were incubated with a block buffer on a rocking platform shaker. Then, samples were incubated with reconstituted mouse cytokine array panel A detection antibody cocktail for 1 h, at +20°C. Then, samples/antibodies mixture was dispensed on the membranes and incubated overnight, at +4°C. Membranes were washed and streptavidin-HRP was added to the membranes for 30 min, at +20°C. After washes, they were incubated with Chemi Reagent Mix and exposed to the chemiluminescence detection using a ChemiDoc XRS+ (Bio-Rad, Hercules, USA) for the imaging. Qualitative images were converted using Image Lab software (Bio-Rad), and quantification of the detected cytokines was assessed as pixel density of dots spotted on the membranes using Image J software and calculated as fold change versus the GFP control group.

**Experiments using iPCS-derived cardiomyocytes**

*Differentiation of iPSC-derived cardiomyocytes*

Cardiomyocytes were cultured and differentiated from induced pluripotent stem cells (iPSC) according to Lemcke et al.^5^ Briefly, iPSCs were cultured on Laminin521 (Biolamina, Sundbyberg, Sweden) coated culture vessels. iPSCs (Takara Bio Inc, Kusatsu, Japan) were maintained in iPS Brew (Miltenyi Biotec, Bergisch Gladbach, Germany), supplemented with Zellshield (Biochrom, Berlin, Germany) at 37°C and 5% CO2. Cardiac differentiation was induced in RPMI 1640 Glutamax media (Thermo Fisher Scientific) containing 1% Zellshield, 1% sodium pyruvate, 200 µM ascorbic acid (all Sigma Aldrich, St. Louis, USA) and 2% B27 without insulin (Miltenyi Biotec) and treated with 1 µM Chir99021, 5 ng/mL basic fibroblast growth factor, 5 ng/mL bone morphogenetic protein 4 and 9 ng/mL Activin A (all Miltenyi Biotec) for three days. Afterward, cells were cultured in RPMI 1640 Glutamax supplemented with B27 with insulin (Miltenyi Biotec) and incubated with 5 mM IWP-2 (Tocris, Bristol, UK) for seven days. Single cells were generated using a dissociation kit (Stemcell Technologies, Vancouver, Canada) according to the manufacturer’s instructions.

*Protein treatment*

After 22-25 days of differentiation, cardiomyocytes were treated with vehicle or recombinant BPIFB4 proteins at a concentration of 20 ng/ml for 7 days. The protein was diluted in medium and renewed with this every two days.

*Detection of Apoptosis*

Following protein treatment, fragmented DNA of cardiomyocytes was labelled using TUNEL assay kit (AATbioquest, Sunnyvale, USA) as specified by the manufacturer. Following DNA labelling, cells were dissociated and measured using BD™ FACS LSRII (Becton Dickinson (BD), Heidelberg, Germany) flow cytometer; at least 10,000 events were acquired. Data analysis was conducted using FACS Diva software (BD, version 6.1.2). Percentages of apoptotic cells were calculated by the inclusion of the singlets (dot plots: SSC-W versus SSC-H; FSC-H vs. FSC-A) and the gate of the positive signals within the dot plot SSC-A versus PE-Cy5 channel (red fluorescence).

*Labelling of mitochondria*

Mitochondria activity of dissociated cardiomyocytes was determined using 300nM Mitotracker deep red FM staining solution (Thermo Fisher Scientific). Labelling was performed as recommended by the manufacturer. Visualization was performed with Zeiss ELYRA LSM 780 (Zeiss) microscope using 40x/NA 1.3 oil objective.

*Evaluation of sarcomere structures*

Dissociated cardiomyocytes were fixed with 2% paraformaldehyde (PFA) for 15 min, followed by incubation with 0.2% Triton-X 100 (all Sigma Aldrich) for 5 min. Fixed cells were labelled with anti-sarcomeric α-actinin (Abcam, ab9465) and goat anti-mouse AlexaFluor 647 secondary antibody (Thermo Fisher Scientific, A-21237). Images were acquired using confocal laser scanning microscopy (ELYRA LSM 780, Zeiss). Z-stacks were generated, and maximum projections were created using Zen software (Zeiss).

Reconstruction of raw images and post-processing was performed with ImageJ software and corresponding plugins. Sarcomere length was evaluated by measuring the distance between neighboring filaments. For the determination of the sarcomere content, binary images were generated and the ridge detection plugin was applied. The filament density was calculated as the percentage of the overall cell area. For the assessment of filament orientation cells were aligned according to their longitudinal axis and orientation was quantified using the directionality plugin.

*Analysis of cell contractility*

To analyze cell contraction, videos of beating cardiomyocytes were recorded using Zeiss ELYRA LSM 780 (Zeiss) microscope for 20 sec; using 4x4 binning and 83Fps. The ImageJ Myocyter plugin was applied to measure contractility of the beating cell clusters [8,9]. Thresholding for the region of interest was performed following pretesting. The user-defined threshold for peak time duration was set to 20%. For each condition, eight videos were analyzed with 8-15 regions of interest examined per condition.

*Immunostaining for BPIFB4*

Cardiomyocytes were fixed with 4% buffered PFA at room temperature and incubated with an antibody for BPIFB4 (1 µg/ml; custom made, Clinisciences, Nanterre, France), overnight at 4°C. Images were taken using an Leica microscope (Leica, Wetzlar, Germany) with a 40× objective.

**Experiments using hcFbs**

*Isolation*

HcFbs were isolated from human auricle fragments obtained from a female patient that underwent cardiac surgical intervention, following written informed consent and in compliance with the Helsinki Declaration upon approval of the local ethical committee IRCCS IEO and Centro Cardiologico Monzino (protocol CCFM C9/607), or female cadaveric donors of Fondazione Banca dei Tessuti di Treviso (MTA n° 257/A1/2016; **Supplementary Table 1**). Cells were isolated and characterized by flow cytometry as previously described [10,11].

*Treatment*

HcFbs were used at the 7th passage for all experiments. HcFbs (2x10^4^) were plated on fibronectin (4 µg/ml, #F1141, MERK, Darmstadt, Germany), starved at 37°C overnight in IMDM supplemented with 1% FBS and treated with recombinant LAV-BPIFB4 protein (20 ng/ml), vehicle (20 ng/ml), TGF-β1 (10 ng/ml; #100-21; Peprotech, Rocky Hill, NJ, USA), or combination of LAV-BPIFB4 protein (20 ng/ml) plus TGF-β1 (10 ng/ml; #100-21; Peprotech, Rocky Hill, NJ, USA) for 24 hours.

*Immunofluorescence*

hcFbs were incubated with an antibody for Collagen I (1 µg/ml; #ab34710, Abcam, Cambridge, UK), Collagen III (1 µg/ml; #ab7778, Abcam), α-SMA (1 µg/ml; #ab5694; Abcam), or BPIFB4 (1 µg/ml; custom made, Clinisciences, Nanterre, France) overnight at 4°C. Images were taken using an Apotome microscope (Zeiss, Oberkochen, Germany) with a 20× objective and quantified with ZEN 2.6 (Blue edition) Carl Zeiss microscopy GmBH 2018 program (Zeiss); positive area (area intensity/µm^2^) was determined in about 50 cells/condition. The experiments were repeated 3 times for each different donor (**Supplementary Table 1**).

**Statistical analyses**

In the *in vitro* and *in vivo* studies, the comparison among groups with one independent variable was performed using a Student’s *t*-test or with the equivalent non-parametric test. When appropriate, one-way ANOVAs (followed by Tukey’s multiple comparisons tests) or Kruskal-Wallis tests (followed by Dunn’s multiple comparison tests) were employed. Comparison among groups with 2 independent variables was performed employing repeated measurements two-way ANOVA followed by Sidak’s multiple comparison test.

In the clinical study, the BPIFB4 values have been transformed due to their extremely right skewed distribution using natural logarithm (Ln) of BPIFB4. Numeric variables distribution was described by median (25^th^, 75^th^ percentiles) and compared among groups by the non parametric two-sided Wilcoxon rank sum test and by the Kruskal Wallis test since Shapiro test p-value was < 0.05 for the analysed variables. The independence between categorical variables was assessed by the Pearson chi square test or by the two-sided Fisher exact test (when at least 20% of the cells reporting the expected frequencies from the Pearson chi square test was < 5 or at least one expected frequency value was < 1). The strength of the correlation between numeric variables was estimated by the Spearman correlation coefficient and corresponding 95% Confidence Interval (95% CI) and by testing the null hypothesis that the true correlation was 0. Logistic regression was used to test for association between BPIFB4 values and binary clinical outcomes with and without adjustment for potential confounders. Potential confounders have been identified as those variables with missing data fraction < 5%, showing evidence of statistically significant correlation with continuous Ln BPIFB4 values (numeric variables)of unbalanced distribution of continuous Ln BPIFB4 across variables’ levels (categorical variables) and evidence of association with three-vessel CAD status.

The significance level has been set to α = 0.05. Statistical analyses have been performed by the R software environment for statistical computing and graphics version 4.0.5 (www.r-project.org) except when specified.

**References**

1. Villa F, Carrizzo A, Spinelli CC, Ferrario A, Malovini A, Maciąg A, et al. Genetic Analysis Reveals a Longevity-Associated Protein Modulating Endothelial Function and Angiogenesis. Circ Res. 2015;117:333-45. doi: 10.1161/CIRCRESAHA.117.305875.
2. Vecchione C, Villa F, Carrizzo A, Spinelli CC, Damato A, Ambrosio M, et al. A rare genetic variant of BPIFB4 predisposes to high blood pressure via impairment of nitric oxide signaling. Sci Rep. 2017;7:9706. doi: 10.1038/s41598-017-10341-x.
3. Katare R, Riu F, Mitchell K, Gubernator M, Campagnolo P, Cui Y, et al. Transplantation of human pericyte progenitor cells improves the repair of infarcted heart through activation of an angiogenic program involving micro-RNA-132. Circ Res. 2011;109:894-906. doi: 10.1161/CIRCRESAHA.111.251546.
4. Avolio E, Meloni M, Spencer HL, Riu F, Katare R, Mangialardi G, et al. Combined intramyocardial delivery of human pericytes and cardiac stem cells additively improves the healing of mouse infarcted hearts through stimulation of vascular and muscular repair. Circ Res. 2015;116:e81-94. doi: 10.1161/CIRCRESAHA.115.306146.
5. Besnier M, Galaup A, Nicol L, Henry JP, Coquerel D, Gueret A, et al. Enhanced angiogenesis and increased cardiac perfusion after myocardial infarction in protein tyrosine phosphatase 1B-deficient mice. FASEB J. 2014;28:3351-61. doi: 10.1096/fj.13-245753.
6. Houssari M, Dumesnil A, Tardif V, Kivelä R, Pizzinat N, Boukhalfa I, et al. Lymphatic and Immune Cell Cross-Talk Regulates Cardiac Recovery After Experimental Myocardial Infarction. Arterioscler Thromb Vasc Biol. 2020;40:1722-1737. doi: 10.1161/ATVBAHA.120.314370
7. Dang Z, Avolio E, Thomas AC, Faulkner A, Beltrami AP, Cervellin C, et al. Transfer of a human gene variant associated with exceptional longevity improves cardiac function in obese type 2 diabetic mice through induction of the SDF-1/CXCR4 signalling pathway. Eur J Heart Fail. 2020;22:1568-1581. doi: 10.1002/ejhf.1840.
8. Grune T, Ott C, Häseli S, Höhn A, Jung T. The "MYOCYTER" - Convert cellular and cardiac contractions into numbers with ImageJ. Sci Rep. 2019;9:15112. doi: 10.1038/s41598-019-51676-x.
9. Lemcke H, Skorska A, Lang CI, Johann L, David R. Quantitative Evaluation of the Sarcomere Network of Human hiPSC-Derived Cardiomyocytes Using Single-Molecule Localization Microscopy. Int J Mol Sci. 2020;21:2819. doi: 10.3390/ijms21082819.
10. Di Maggio S, Milano G, De Marchis F, D'Ambrosio A, Bertolotti M, Palacios BS, et al. Non-oxidizable HMGB1 induces cardiac fibroblasts migration via CXCR4 in a CXCL12-independent manner and worsens tissue remodeling after myocardial infarction. Biochimica et Biophysica acta. Molecular Basis of Disease. 2017 Nov;1863(11):2693-2704. DOI: 10.1016/j.bbadis.2017.07.012. PMID: 28716707
11. Scavello F, Zeni F, Milano G, Macrì F, Castiglione S, Zuccolo E, et al. Soluble Receptor for Advanced Glycation End-products regulates age-associated Cardiac Fibrosis. Int J Biol Sci. 2021 Jun 11;17(10):2399-2416. doi: 10.7150/ijbs.56379. PMID: 34326683; PMCID: PMC8315019.

**Supplementary Figure 1.** **Mouse cytokine arrays.** Qualitative analysis of inflammatory cytokines in plasma collected from mice with myocardial infarction after gene therapy with AAV-LAV-BPIFB4 or GFP control. n=4/group. Targets are reported in the legend next to the membranes.

**Supplementary Figure 2.** **BPIFB4 localization in iPSC-derived cardiomyocytes**. Representative fluorescence images of cardiomyocytes exposed to recombinant LAV-BPIFB4 protein or vehicle and immunolabelled with the polyclonal antibody to the BPIFB4 N-terminus (red colour). Nuclei are identified by Hoechst staining (blue labelling). Scale bars: 20 μm.

**Supplementary Figure 3.** **LAV-BPIFB4 blunts the TGF-β1-induced pro-fibrotic response.** HcFbs were stimulated with the recombinant LAV-BPIFB4 protein, Vehicle, TGF-β1 or combination of LAV-BPIFB4 protein plus TGF-β1. In the left panel, representative images of Collagen I and α-SMA stained in green; nuclei were stained with Hoechst (blue). Bar scale, 50 μm. In the right panel, quantification of Collagen I and α-SMA expression. Bar graphs represent mean ± SD (n=3). Data were analyzed using two-sided t-test for paired samples.

**Supplementary Figure 4.** **BPIFB4 localization in cardiac fibroblasts.** Representative fluorescence images of hcFbs exposed to recombinant LAV-BPIFB4 protein or vehicle in the presence and absence of TGF-β1, and immunolabelled with the polyclonal antibody to the BPIFB4 N-terminus (red colour). Nuclei are identified by Hoechst staining (blue labelling). Scale bars: 50 μm.

**Supplementary Table 1. Patient information of derived hcFbs.**

**Supplementary Table 2.** **Antibodies and experimental conditions employed for immunohistochemical analyses on mouse sections.**
