# Supplementary figures and images for: BPIFB4 and its longevity-associated haplotype protect from cardiac ischemia in humans and mice
Source: Cell Death Dis. 2023 Aug 15;14(8):523. doi: 10.1038/s41419-023-06011-8 (PMC10427721; doi:10.1038/s41419-023-06011-8)

## Slide 1
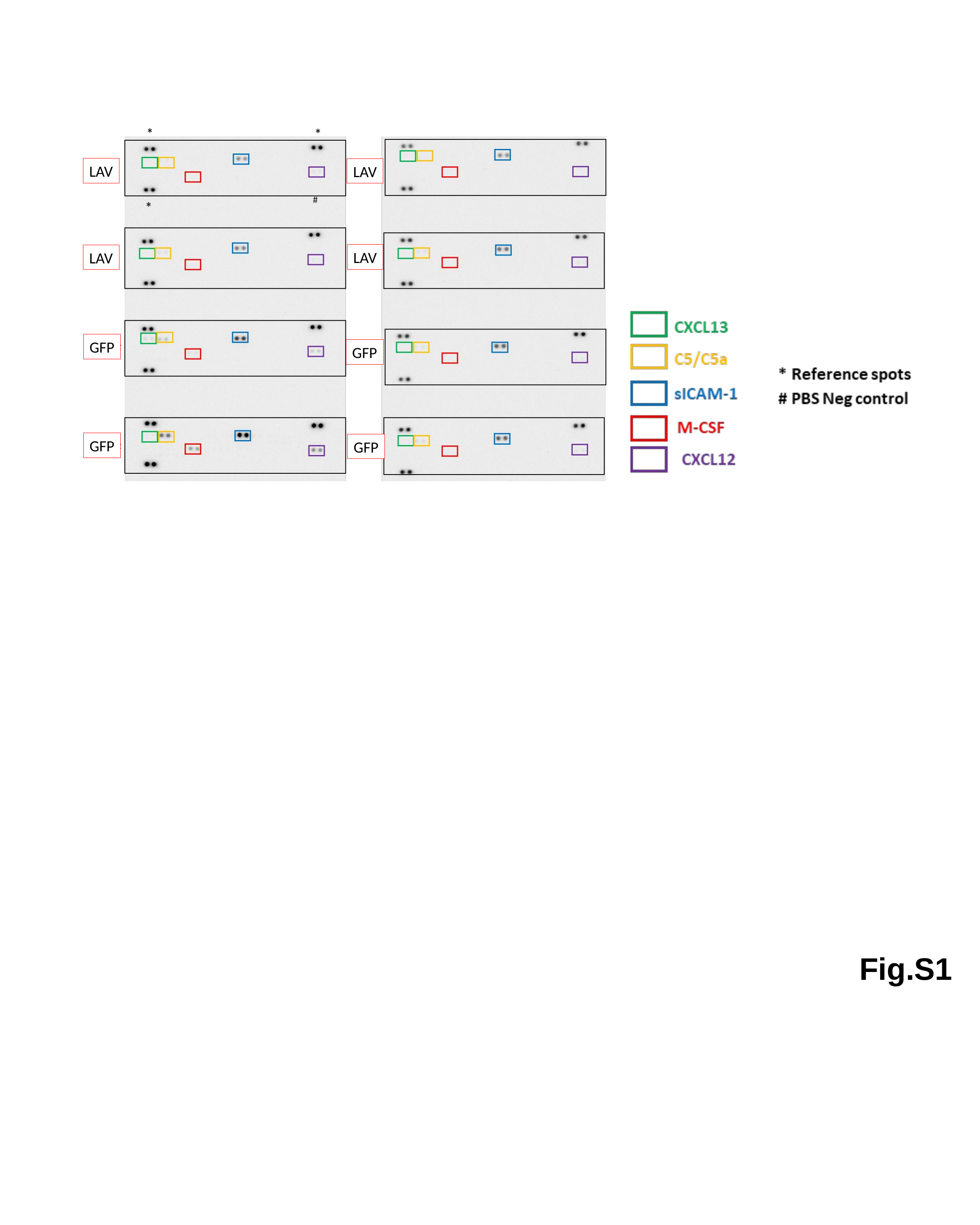

LAV
LAV
LAV
LAV
GFP
GFP
GFP
GFP
Fig.S1

Supplement: Supplementary file 2 — Supplementary Figure 1 [file 41419_2023_6011_MOESM2_ESM.pptx]

## Slide 1
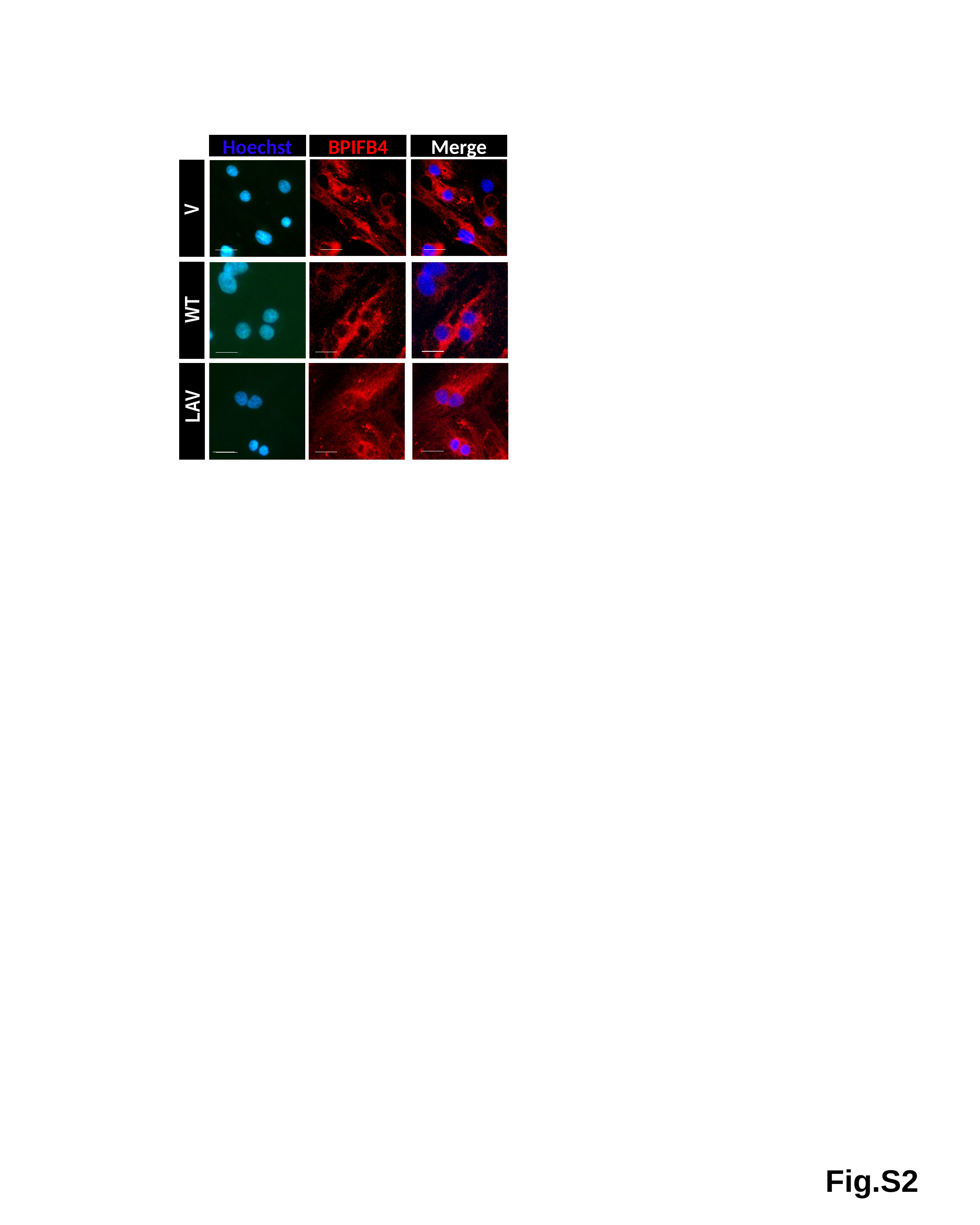

Merge
Hoechst
BPIFB4
V
WT
LAV
Fig.S2

Supplement: Supplementary file 3 — Supplementary Figure 2 [file 41419_2023_6011_MOESM3_ESM.pptx]

## Slide 1
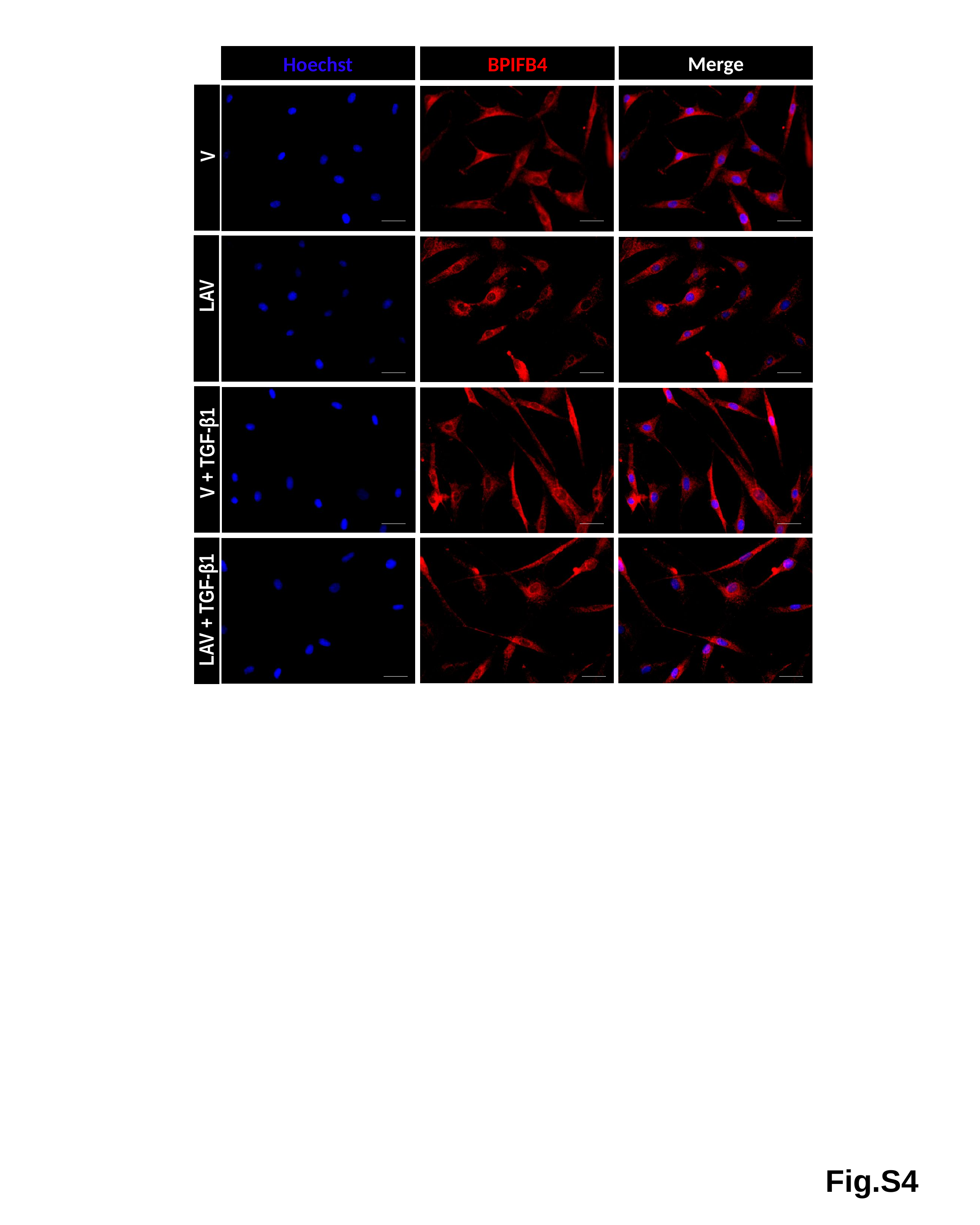

Merge
Hoechst
BPIFB4
V
LAV
V + TGF-β1
LAV + TGF-β1
Fig.S4

Supplement: Supplementary file 5 — Supplementary Figure 4 [file 41419_2023_6011_MOESM5_ESM.pptx]
