# Supplementary material for: BPIFB4 and its longevity-associated haplotype protect from cardiac ischemia in humans and mice
Source: Cell Death Dis. 2023 Aug 15;14(8):523. doi: 10.1038/s41419-023-06011-8 (PMC10427721; doi:10.1038/s41419-023-06011-8)
Supplement: Supplementary file 4 — Supplementary Figure 3 [file 41419_2023_6011_MOESM4_ESM.pptx]

## Slide 1
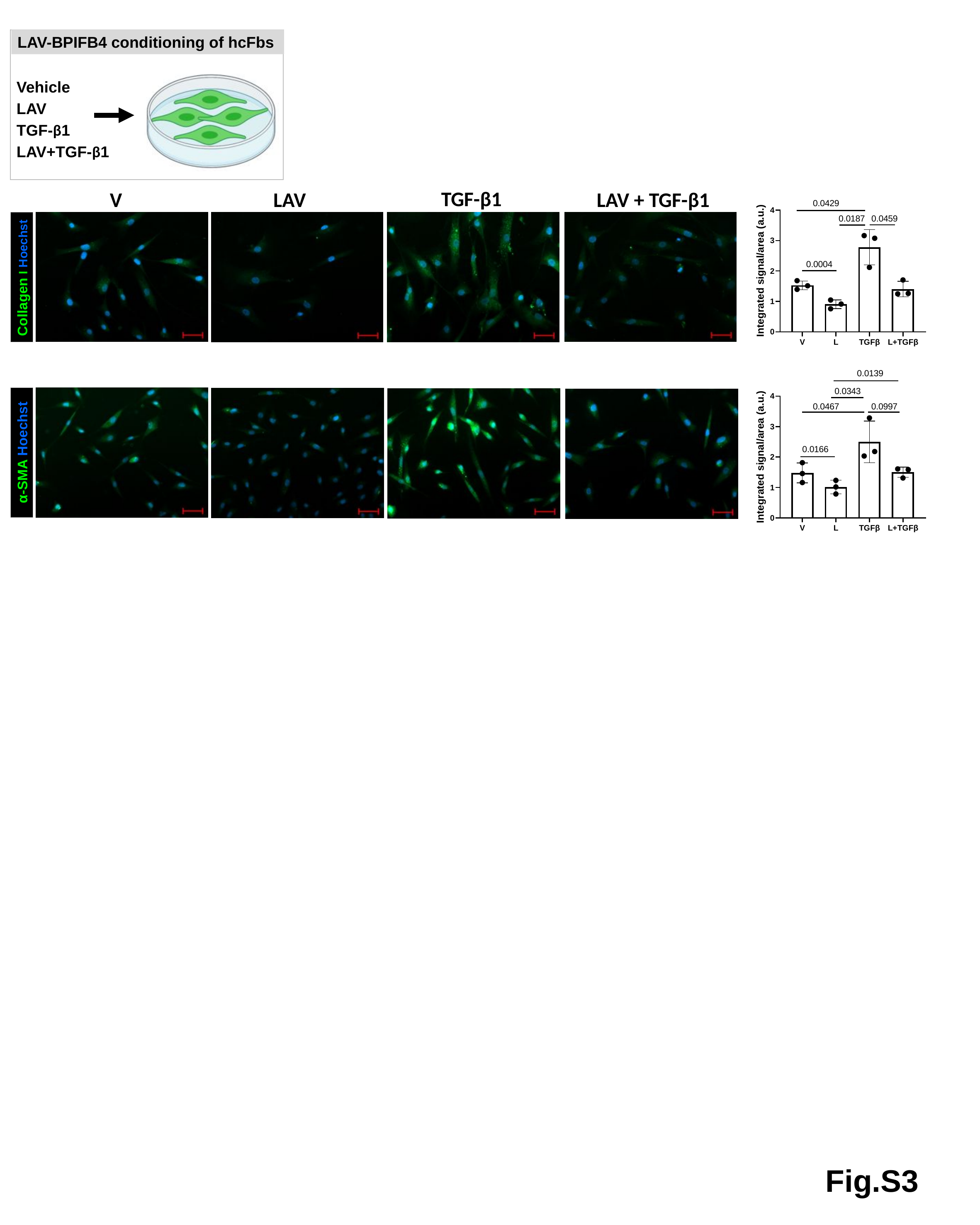

LAV-BPIFB4 conditioning of hcFbs
Vehicle
LAV
TGF-β1
LAV+TGF-β1
TGF-β1
V
LAV
LAV + TGF-β1
Collagen I Hoechst
α-SMA Hoechst
Fig.S3
