# Supplementary material for: BPIFB4 and its longevity-associated haplotype protect from cardiac ischemia in humans and mice
Source: Cell Death Dis. 2023 Aug 15;14(8):523. doi: 10.1038/s41419-023-06011-8 (PMC10427721; doi:10.1038/s41419-023-06011-8)
Supplement: Supplementary file 6 — Supplementary Table 1 [file 41419_2023_6011_MOESM6_ESM.docx]

**Supplementary Table 1**

| Patient  ID | Age  (years) | Gender | History | CD44, CD29, CD73, CD105, CD90 | CD45, CD14, CD34, CD31, HLA-DR |
| --- | --- | --- | --- | --- | --- |
| #1 | 34 | F | Healthy | + | - |
| #2 | 50 | F | Healthy | + | - |
| #3 | 74 | F | Valvular Heart Disease | + | - |
