# Supplementary material for: BPIFB4 and its longevity-associated haplotype protect from cardiac ischemia in humans and mice
Source: Cell Death Dis. 2023 Aug 15;14(8):523. doi: 10.1038/s41419-023-06011-8 (PMC10427721; doi:10.1038/s41419-023-06011-8)
Supplement: Supplementary file 7 — Supplementary Table 2 [file 41419_2023_6011_MOESM7_ESM.docx]

**Supplementary Table 2.**

| Antigen | Company (Catalog N°) | Dilution | Antigen retrieval | Incubation time and temperature | Secondary antibody | Incubation time |
| --- | --- | --- | --- | --- | --- | --- |
| α-Sarcomeric Actin | SIGMA (A2172) | 1:400  or 1:200 | Citric buffer (pH6), 98°C, 40’  or none | 1h, 37°C  or 2h, RT  or O/N, 4°C | Cy5 1:400  or Alexa Fluor 647 1:200  or TRITC 1:200 | 1h, 37°C  or 1h, RT |
| isolectin gs-Ib_4_-biotinILATED | Life Technologies (121414) | 1:200 | Citric buffer (pH6), 98°C, 30’  or none | O/N, 4°C | Streptavidin- Alexa Fluor 488 1:200 | 1h, RT |
| Legend: |  | | | | | |
| A488 | Alexa Fluor 488 labeled donkey Antibody OR streptavadin | | | | | |
| A555 | Alexa Fluor 555 labeled donkey Antibody | | | | | |
| A568 | Alexa Fluor 568 labeled donkey OR goat Antibody | | | | | |
| A633 | Alexa Fluor 633 labeled donkey Antibody | | | | | |
| A647 | Alexa Fluor 647 labeled donkey OR goat Antibody | | | | | |
| TRITC | TRITC labeled goat Antibody | | | | | |
| Cy5 | Cy5 labeled donkey Antibody | | | | | |
| IHC | Envision Detection system Peroxidase/DAB (Agilent/DAKO), Rabbit/Mouse | | | | | |
